# Glycolysis Paradigm Shift Dictates a Reevaluation of Glucose and Oxygen Metabolic Rates of Activated Neural Tissue

**DOI:** 10.3389/fnins.2018.00700

**Published:** 2018-10-10

**Authors:** Avital Schurr

**Affiliations:** Department of Anesthesiology and Perioperative Medicine, School of Medicine, University of Louisville, Louisville, KY, United States

**Keywords:** cerebral metabolic rate for glucose, cerebral metabolic rate for oxygen, glycolysis, lactate, mitochondrial lactate dehydrogenase, polarogaphy, BOLD fMRI, paradigm shift

## Abstract

In 1988 two seminal studies were published, both instigating controversy. One concluded that “the energy needs of activated neural tissue are minimal, being fulfilled via the glycolytic pathway alone,” a conclusion based on the observation that neural activation increased glucose consumption, which was not accompanied by a corresponding increase in oxygen consumption (Fox et al., 1988). The second demonstrated that neural tissue function can be supported exclusively by lactate as the energy substrate (Schurr et al., 1988). While both studies continue to have their supporters and detractors, the present review attempts to clarify the issues responsible for the persistence of the controversies they have provoked and offer a possible rationalization. The concept that lactate rather than pyruvate, is the glycolytic end-product, both aerobically and anaerobically, and thus the real mitochondrial oxidative substrate, has gained a greater acceptance over the years. The idea of glycolysis as the sole ATP supplier for neural activation (glucose → lactate + 2ATP) continues to be controversial. Lactate oxidative utilization by activated neural tissue could explain the mismatch between glucose and oxygen consumption and resolve the existing disagreements among users of imaging methods to measure the metabolic rates of the two energy metabolic substrates. The postulate that the energy necessary for active neural tissue is supplied by glycolysis alone stems from the original aerobic glycolysis paradigm. Accordingly, glucose consumption is accompanied by oxygen consumption at 1–6 ratio. Since Fox et al. (1988) observed only a minimal if non-existent oxygen consumption compared to glucose consumption, their conclusion make sense. Nevertheless, considering (a) the shift in the paradigm of glycolysis (glucose → lactate; lactate + O_2_ + mitochondria → pyruvate → TCA cycle → CO_2_ + H_2_O + 17ATP); (b) that one mole of lactate oxidation requires only 50% of the amount of oxygen necessary for the oxidation of one mole of glucose; and (c) that lactate, as a mitochondrial substrate, is over eight times more efficient at ATP production than glucose as a glycolytic substrate, suggest that future studies of cerebral metabolic rates of activated neural tissue should include along with the measurements of CMR_O_2__ and CMR_glucose_ the measurement of CMR_lactate_.

## Introduction

**FIGURE 1:**
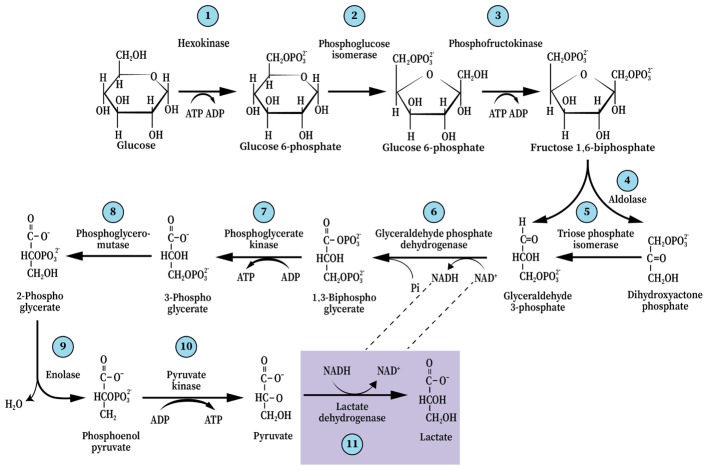
The glycolytic pathway and the 10 enzymatic steps that break down one molecule of glucose into two trios molecules, ending with pyruvate (aerobically) or, with an 11th step, lactate (anaerobically). This dogmatic configuration has guided scientists and science students ever since it was elucidated in 1940.

The term “glycolysis” is almost synonymous with the term “biochemistry,” as the former is responsible for the birth of the latter. The elucidation of the glycolytic pathway in 1940 was such a great leap of progress in our understanding of the basic cellular processes of life that less than a decade later it was already being taught universally as part of every high school biology curriculum. If there were ever a scientific dogma to withstand the test of time, glycolysis is it. In principle, the 1940 map of the glycolytic pathway and its ten steps, as illustrated by Gustav Embden, Otto Meyerhof, and Jakub Karol Parnas is the same map one finds today in every biochemistry, physiology or neuroscience textbook or any website dealing with the topic. Surely, details have been added over the years, both in terms of the structure of the different players at each of the pathway steps, their mechanism of function and their regulation, but the ten steps and their order remained unchanged (**Figure 1**). Also in 1940, an addendum to glycolysis was created with an eleventh step, according to which, it takes place only under anaerobic conditions (**Figure 1**). This step involves the conversion of the (aerobic) end-product of glycolysis, pyruvate, to lactate. This conditional step, in essence, splits the pathway into two types, an aerobic and an anaerobic glycolysis. Curiously, despite the great advances in our knowledge and understanding of enzyme structure and function, no mechanism, enzymatic or other, has been offered to explain how the presence of oxygen prevents the conversion of pyruvate to lactate or conversely, how the absence of oxygen “catalyzes” that conversion.

**FIGURE 2:**
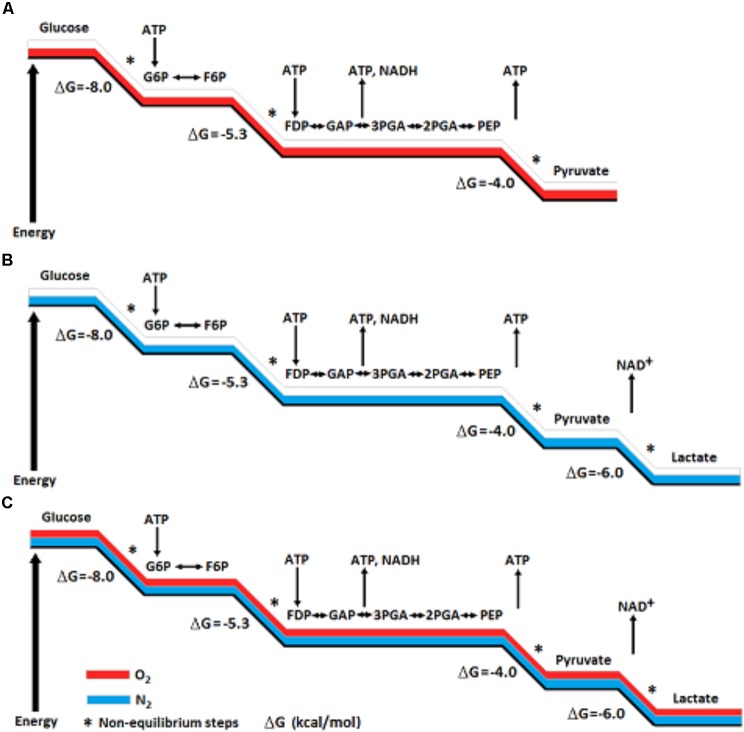
A Schematic presentation of potential free-energy change profile of aerobic glycolysis **(A)** and of anaerobic glycolysis **(B)**. The potential free-energy change of pyruvate conversion to lactate dictates that this reaction should proceed regardless of the oxygenation conditions **(C)**. The conversion of pyruvate to lactate also assures the continuous supply of NAD^+^, an essential component for the cyclical nature of the glycolytic pathway.

Yet, the ability of red blood cells (RBC), the richest tissue in oxygen content, to produce lactate glycolyticly is accepted unquestioningly. Could the absence of mitochondria in RBC explain that production? Will adding isolated mitochondria to RBC suspension change the glycolytic production of lactate to pyruvate? Nevertheless, scientists in every field that deals with energy metabolism, directly or indirectly, accept the concept of glycolytic duality – two different outcomes, aerobic, where pyruvate is the end-product, and anaerobic, where lactate is the end-product. However, by arbitrarily determining that aerobic glycolysis ends with the production of pyruvate, the need for a renewed supply of NAD^+^ was ignored. This need is at the basis of glycolysis cyclical nature and is achieved by the conversion of pyruvate and NADH to lactate and NAD^+^, respectively (**Figure 1**). Also, the standard free-energy (ΔG^0′^) change of pyruvate conversion to lactate (−6 kcal/mol), means that this conversion should ensue independently of the presence or absence of oxygen; As can be seen in **Figure 2A**, the free-energy change profile of (aerobic) glycolysis ends with the conversion of phosphoenolpyruvate to pyruvate, even though the potential free-energy change of pyruvate conversion to lactate (anaerobic glycolysis, **Figure 2B**) dictates that this reaction should proceed regardless of the oxygenation conditions (**Figure 2C**).

In addition to the requirement for glycolytic NAD^+^ replenishment and the free-energy change potential of the pyruvate to lactate conversion, there are other considerations that should lead one to question the original 10-step configuration of glycolysis. Moreover, the high affinity of pyruvate to the cytosolic enzyme lactate dehydrogenase (c-LDH) should not allow for enough free pyruvate that is necessary to drive the mitochondrial tricarboxylic acid (TCA) cycle. This high affinity may also explain the fact that the normal lactate/pyruvate ratio in blood and other tissues is >10 (Havel et al., 1950), a value that cannot correspond with the proposal of pyruvate as the end-product of glycolysis under normal (aerobic) conditions. Even more important are the numerous studies published over the past 30 years, experimentally pointing at lactate as the real end-product of glycolysis. Clearly, these points do not in any way argue against the ability of mitochondria to uptake and utilize pyruvate as a substrate for the TCA cycle, but high cellular lactate/pyruvate ratio indicates that it is not a major one. All the same, despite the continuous accumulation of experimental evidence in support of lactate as the glycolytic end-product, aerobically and anaerobically, the paradigm of glycolytic duality persists in textbooks, classrooms, and citations in the most recent published papers (Yellen, 2018).

To understand this persistence, one must consider the history of scientific paradigms and revolutions (Kuhn, 1996). Evidently, such persistence in embracing the established, albeit flawed, paradigm is a well described and recognized phenomenon, which Margolis (1993) described as “habit of mind,” a term that *“suggests entrenched responses that ordinarily occur without conscious attention, and that even if noticed are hard to change.”* (See also Schurr, 2014).

Where the glycolytic pathway is concerned, one must delve into the literature published in the decades preceding its decipherment in 1940. In his excellent essay, Margolis explains how “habits of mind” can and do govern scientific beliefs. At times, these habits form a barrier, which blocks or delays the acceptance of a new idea that could lead to a paradigm shift. Additionally, one may argue that at times, the scientists who established a given scientific concept and/or their followers actively head the efforts against the eventual shift. Nonetheless, the established conception of glycolysis is at the basis of how we understand and interpret glucose and oxygen CMR studies both during rest and activation. As is detailed below, those studies are sometime at odds with each other. It precedes by a short review of the history of glycolysis, highlighting chronologically the key discoveries and concepts that have led to the establishment of its classical understanding and the more recent studies that have led to questioning that established dogma. A more detailed review of that history is available (Schurr, 2014; Rogatzki et al., 2015).

## Glycolysis Circa 1940: Evidence, Considered and Overlooked, Conjecture, and Belief

Two major factors have been most likely responsible for glycolysis’ “split personality.” The first was the negative reputation assigned to lactate. From the outset, upon its discovery in spoiled milk by Carl Wilhelm Scheele in 1780, and until the early 1990s, as exemplified by the lactic acidosis hypothesis (Siesjö, 1981), lactate was assumed to be a culprit. Siesjo’s lactic acidosis hypothesis postulated lactate to be responsible for delayed ischemic neuronal damage, where the monocarboxylate was portrayed as a waste product at best and poison at worse. Recently, many of the studies published during the first four decades of the 20th century that established and promoted the lactate’s negative reputation were reviewed (Schurr, 2014). This reputation has prevented any attempt to bring up a possible role of importance for this molecule beyond it being a waste product that must be either disposed of or be recycled. Therefore, for glycolysis to play a key role in energy metabolism by producing adenosine triphosphate (ATP), while simultaneously providing the main aerobic substrate, pyruvate, for the TCA cycle, assigning such a role to lactate was inconceivable. The most telling of that attitude was exhibited by E. G. Holmes, B. D. Holmes, and C. A. Ashford who, between 1925 and 1933, had demonstrated in several outstanding studies that cerebral tissue is capable of oxidizing lactate. Hence, the evidence regarding a possible utilization of lactate as energy substrate was ignored by these scientists and by the scientific community of the day (Holmes and Holmes, 1925, 1926, 1927; Ashford and Holmes, 1929; Holmes and Ashford, 1930; Holmes, 1930, 1932, 1933). Holmes, Holmes, and Ashford, being members of a scientific community that believed lactate to be a waste product, could not bring themselves to consider the possibility that its oxidation may signal anything, but a disposal reaction. The second factor was the assumption laid down by Krebs and Johnson (1937) according to which pyruvate is the substrate of the TCA cycle. Krebs and Johnson published their seminal results 3 years prior to the decipherment of the glycolytic sequence of reactions, where they surmised, albeit with a question mark, that pyruvate is the substrate that enters the TCA cycle. It is easy to imagine how the elucidators of the glycolytic pathway, relying on Krebs and Johnson’s postulate, made, what appears to be a conjecture, and decided that since pyruvate is the substrate of the TCA cycle it must also be the glycolytic aerobic end-product.

As to the barrier Holmes, Holmes, and Ashford faced during their period, they had clearly accepted the prevailing notion that existed in their time, i.e., that lactate is a waste product of anaerobic glucose utilization, a notion that preceded any clear knowledge and understanding of the glycolytic enzymatic reactions. The two overshadowing figures of the period were A. V. Hill and O. Meyerhof, both Nobel laureates of the 1920s and both leaders in the field of muscular tissue energy metabolism. These two giants and their research groups had established the prevailing standard of the day that “lactate is a waste product of glucose oxidation.” Lactate oxidation could have never been perceived as a reaction that may have any other purpose than disposal of waste. Under such circumstances the most Holmes, Holmes, and Ashford could expect was that their findings would be accepted as a real reaction, possibly unique to brain tissue that is aimed at lactate elimination. Nowhere in their papers did these authors ever consider such a reaction to be anything else, which would explain why their elegant studies faded away without further pursuit. H. A. Krebs received his Nobel Prize in Physiology or Medicine in 1953 for his contribution to the exposition of the TCA cycle, which no doubt had strengthened the assumption that pyruvate must be the end product of aerobic glycolysis and the substrate of the TCA cycle. However, the original glycolytic pathway (aerobic) that ends with pyruvate does not offer a biochemical solution for the renewal of reducing equivalents, i.e., how NADH is oxidized back to NAD^+^. To overcome this deficiency, a search for alternative processes ensued where the malate-aspartate shuttle and the glycerol phosphate shuttle were proposed (Berry, 1971; Safer et al., 1971; Kauppinen et al., 1987; Ramos et al., 2003; McKenna et al., 2006; Contreras and Satrustegui, 2009; Gellerich et al., 2012).

## Glycolysis Circa 1990: New Discoveries, Entrenchment, Resistance, and “Habit of Mind”

The now defunct lactic acidosis hypothesis of delayed ischemic neuronal damage (Kalimo et al., 1981; Rehncrona et al., 1981; Siesjö, 1981) was heavily promoted and considered to be an excellent working hypothesis throughout the 1980s and 1990s. Thus, for more than four decades after the glycolytic pathway was revealed, lactate’s harmful reputation lingered, persuading most scientists working in the field to accept it as the culprit of delayed neuronal damage observed post-cerebral ischemia. Brooks (1985) demonstrated that in exercising muscle lactate is both a glycolytic product and an oxidative substrate. He postulated the existence of an intracellular lactate shuttle between the cytosol, where glycolytic lactate is produced, and the mitochondrion, where lactate is oxidatively consumed as it enters this organelle. Such mechanism would required the presence of LDH within the mitochondrial membrane (m-LDH), the enzyme that converts lactate to pyruvate, the monocarboxylate that eventually, enters the TCA cycle. Several years later Brooks et al. (1999a,b) demonstrated the presence of both a monocarboxylate transporter 1 (MCT1) and a LDH in mitochondria. Fox and Raichle (1986) showed a focal physiological uncoupling between cerebral blood flow (CBF) and oxidative metabolism in response to somatosensory stimulation in humans and in Fox et al. (1988) demonstrated that during focal physiologic neural activity the consumption of glucose is non-oxidative (Warburg effect), assuming glycolytic ATP and lactate production. Shortly thereafter, Schurr et al. (1988) observed the ability of lactate to maintain normal neuronal function *in vitro* in the absence of glucose or any other energy substrate. As the number of studies and reviews that support lactate role in oxidative energy metabolism in muscle (Brooks, 1998, 2000, 2002a,b; Brooks et al., 1999a,b) and brain (Izumi et al., 1994; Pellerin and Magistretti, 1994; Larrabee, 1995, 1996; Tsacopoulos and Magistretti, 1996; Hu and Wilson, 1997; Schurr et al., 1997, 1999a,b; Schurr and Rigor, 1998; Magistretti and Pellerin, 1999; Magistretti et al., 1999; Magistretti, 2000; Qu et al., 2000; Van Hall, 2000; Bliss and Sapolsky, 2001; Bouzier-Sore et al., 2003; Mangia et al., 2003; Smith et al., 2003; Dalsgaard et al., 2004; de Bari et al., 2004, 2010; Kasischke et al., 2004; Aubert et al., 2005; Schurr, 2006; Atlante et al., 2007; Schurr and Payne, 2007; Passarella et al., 2008; Herrero-Mendez et al., 2009; Zielke et al., 2009; Schurr and Gozal, 2011; Sotelo-Hitschfeld et al., 2012; Barros, 2013; Barros et al., 2013; Schurr, 2014; Rogatzki et al., 2015; Mächler et al., 2016; Barros and Weber, 2018; Barros et al., 2018) increased, the resistance to this concept escalated. As to muscle oxidative lactate utilization and the role of m-LDH in it, the pushback was based on the argument that mitochondria do not contain LDH (Rasmussen et al., 2002; Sahlin et al., 2002). Where brain oxidative lactate utilization was concerned, the pushback relied on the unfounded assumption that those who support the role of lactate as an oxidative energy substrate promote the idea that this monocarboxylate is, potentially, an alternative substrate able to replace glucose (Chih et al., 2001; Dienel and Hertz, 2001, 2005; Chih and Roberts, 2003; Hertz, 2004; Hertz and Dienel, 2005; Hertz et al., 2007; Dienel, 2012a,b). Accordingly, lactate was portrayed as an impossible competitor of glucose and several studies were designed to demonstrate the obligatory role of the latter as the energy substrate that maintains neuronal functions (Dienel and Cruz, 2004; Fillenz, 2005; Bak et al., 2006; Cruz et al., 2007; Gandhi et al., 2009). Throughout the 1990s, Siesjo and his followers were at the forefront, resisting the postulated role of lactate in energy metabolism, using the lactic acidosis hypothesis of delayed neuronal ischemic damage as the flag of that resistance. Where the obligatory role of glucose as the main energy metabolic substrate is concerned, this role has never been disputed by those who demonstrated lactate oxidative utilization as an energy substrate. The role of lactate should have been seen as the most plausible outcome of glucose breakdown via the glycolytic pathway, where lactate, not pyruvate, is the substrate of the mitochondrial TCA cycle (Schurr, 2014). That skepticism most likely originated in “habit of mind” (Margolis, 1993) and a possible existence of a barrier, which prevents one from accepting the necessary shift in paradigm.

## Lactate is Always the End-Product of Glycolysis

As mentioned earlier, support for the initial discoveries of Brooks (1985) and Schurr et al. (1988) has been provided by multiple studies published in the 1990s and the early 2000s. Yet, the old dogma of separated aerobic and anaerobic glycolysis has persisted. Despite the publication of studies that appear to sway the balance toward acceptance of a paradigm shift, it seems that the barrier has been too high for some to overcome. Nevertheless, in a recent review paper (Hertz et al., 2014) some acceptance of lactate as an important energetic molecule is evident, though the barrier for acceptance of it as the main end-product of glycolysis still exists, as the authors chose not to cite in that review any of the numerous studies that clearly demonstrate lactate to be the real glycolytic end-product. Understandably, the push for changing a scientific standard is usually being led by the very scientists who have performed the experiments that prompted them to question the existing one. They are the ones who would check and recheck themselves and their experimental design over and over again, because their original results did not fit the existing paradigm. The first instinct of any scientist who faces an experimental outcome that does not agree with the norm is to question the outcome itself, not the norm. However, when such an outcome not only repeats itself, but more importantly, is confirmed by other scientists, then the existing standard must be questioned. This is especially true when the support for a shift originates from laboratories that use different experimental models and approaches, forming a more encompassing and general concept, which is much more difficult to reject. Eventually, once a shift has taken place, an examination is required of additional processes that have been neatly linked to the original paradigm, links that could be weakened or cannot longer exist as originally described. In the case of glycolysis with lactate as its end-product and the traditional link via pyruvate to the mitochondrial TCA cycle, that link cannot exist under the new concept. The existence of m-LDH has been established by numerous studies and therefore, the conversion of lactate to pyruvate most likely takes place in the mitochondrion (Brooks et al., 1999a,b; Valenti et al., 2002; de Bari et al., 2004, 2010; Atlante et al., 2007; Hashimoto and Brooks, 2008; Hashimoto et al., 2008; Passarella et al., 2008, 2014; Pizzuto et al., 2012; Elustondo et al., 2013). Accordingly, lactate, the end-product of glycolysis, is also the real substrate of the mitochondrial TCA cycle (lactate → pyruvate → acetyl-CoA → TCA).

Consequently, glycolysis’ paradigm shift should compel one to reconsider the findings of Fox et al. (1988) and their interpretation, including those of many other ensuing studies with similar interpretation. As to the role of the malate-aspartate shuttle in a paradigm where lactate is always the glycolytic end-product and the initial mitochondrial substrate for the TCA cycle, Kane (2014) offered and elegant postulate according to which the two mechanisms are not exclusive of each other.

## Glucose, Lactate, and Oxygen Consumption of Activated Neural Tissue

Stimulation-induced increase in brain activity must be supported by an increase in energy supply. This relationship has always been believed to be true and assumed to be fulfilled by an increase in supply of both glucose and oxygen through the elevation in CBF, glycolytic flux, and mitochondrial respiration. In theory, this basic concept of the aerobic conversion of glucose to energy requires, if it is to proceed to completion, six moles of oxygen per each mole of glucose. Measurements of both glucose and oxygen consumption in the brain *in vivo* have been available for over four decades. Tracer methods were developed for these measurements, using isotopes such as ^15^O, ^14^C, ^13^C, and ^18^F. In practice, the oxygen to glucose ratio under resting conditions was regularly measured to be significantly lower than 6:1, a discrepancy believed to occur due to glucose utilization in other metabolic activities that do not require oxygen. Consequently, measurements of cerebral metabolic rate of oxygen (CMR_O_2__) and glucose (CMR_glucose_) frequently yield ratios of <5:1. The discovery by Fox et al. (1988) that physiological stimulation significantly increased both CBF and CMR_glucose_, while CMR_O_2__ was minimally increased or not at all, had dumbfounded many and have challenged investigators to provide a mechanistic explanation to such a phenomenon. The conclusion reached by Fox et al. (1988) was that “energy expenditures of neural activity are far less than has been inferred from the large increases in glucose uptake…” This conclusion was based on the assumption that glucose consumption, when not accompanied by a corresponding oxygen consumption means that glycolysis is not coupled to mitochondrial respiration and that the minute amount of ATP produced via the glycolytic pathway (2 mol of ATP per mol of glucose) is sufficient to support the increased energy demands of stimulated neural tissue. The fact that cerebral tissue is capable of utilizing lactate as an energy substrate, as detailed in the previous sections of this review, has presented a serious problem to many scientists in the field, since it contradicted the prevailing dogma, according to which, lactate is a useless end-product of glycolysis. Consequently, where CMR_glucose_ and CMR_O_2__ are concerned, multiple studies either discounted the possible role lactate plays as energy requirements increase upon stimulation or completely ignore such role. Consequently, misinterpretation of CMR_glucose_ and CMR_O_2__ measurements could lead to the wrong conclusions about the mechanism by which energy requirements of the stimulated neural tissue are being fulfilled. For instance, Fox et al. (1988) concluded from their results that physiological stimulation requires less energy than previously believed and that glucose consumption induced by transient increases in neural activity is in access of that consumed by oxidative metabolism. Others have used the term “aerobic glycolysis” to signify that neural activity acquires its energy needs from glycolysis alone, despite the presence of oxygen (Warburg Effect). The use of the term “aerobic glycolysis” confused its meaning with the one used to describe the conversion of glucose to pyruvate and the utilization of the latter in the mitochondrial TCA cycle according to the original paradigm of aerobic glycolysis. In contrast to the study by Fox et al. (1988), Hyder et al. (1997) measured CMR_glucose_ and CMR_O_2__ of stimulated somatosensory cortex in anesthetized rats employing ^1^H, ^13^C NMR and the calculated ratio of glucose to oxygen utilized in every rat used in the study was approximately 6:1. These values and those published by Ueki et al. (1988) disagree with the conclusions of Fox et al. (1988). Such disagreement may arise from differences in measurement techniques, animal or human subjects, brain area of stimulation or unbefitting assumptions and postulations.

## Direct and Indirect Measurements of CMR_glucose_ and CMR_O_2__

Fox et al. (1988) titled their study “Non-oxidative glucose consumption during focal physiologic neural activity.” Employing ^18^F-labeled 2-fluoro-2-deoxy-D-glucose to measure CMR_glucose_, a method originally developed over a decade earlier (Sokoloff et al., 1977), and ^15^O-labeled molecular O_2_ to measure CMR_O_2__, the investigators stated that transient increases in neural activity increase glucose tissue uptake in excess of that consumes by oxidative metabolism. They concluded these findings to indicate that neural activity consume much less energy than previously believed. Moreover, since a corresponding increase in CBF was also detected, they stated that the reason for this increase is for purposes other than oxidative metabolism. These conclusions stemmed from the prevailing postulate that over 90% of glucose consumption of the resting brain is oxidative and less than 5% of that consumption ends in glycolytic lactate production. Since oxidative consumption of 1 mol of glucose produces approximately 34 mol of ATP, while glycolytic consumption of 2 mol of glucose produces only 2 mol of ATP, it can be easily calculated that oxidative consumption of glucose is responsible for almost 100% of the resting brain ATP production. The discovery by Fox et al. (1988) that brain stimulation increases glucose consumption without a corresponding increase in oxygen consumption shook the established belief according to which increased brain activity requires increased energy supply. Nevertheless, following the publication of this seminal paper, the laboratory of Marcus Raichle has become a leading center for functional brain imaging Raichle (2009). Imaging technologies, beginning with x-ray computed tomography (CT), through positron emission tomography (PET), near-infrared spectroscopy (NIRS), and more recently magnetic resonance imaging (MRI), have become the tools of choice for measuring brain metabolism during rest and activity. Today, the most popular technology for these purposes is the blood oxygen level dependent (BOLD) functional magnetic resonance imaging (fMRI) developed by Ogawa et al. (1990). In principle, BOLD fMRI measures changes in blood oxygenation as they relate to brain activity, however, that relationship is somewhat obscure mainly because no direct neural activity is being measured. Electrophysiology does allow for direct measurement of neural activity and when combined with direct oxygen concentration measurements, such as oxygen polarography, it also provides higher resolution than BOLD fMRI (Bentley, 2014). Similarly to the use of an oxygen electrode for measurements of tissue oxygen concentration, glucose, and lactate electrodes can also be used to measure local tissue concentrations of the hexose and the monocarboxylate.

**FIGURE 3:**
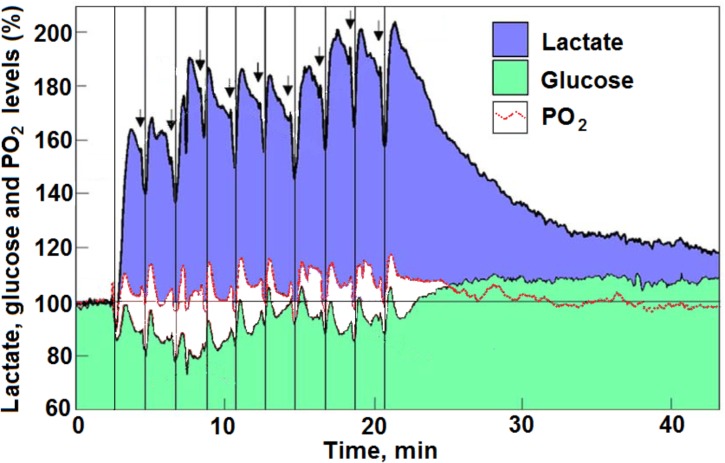
Profiles of time course and dynamic relationships of local extracellular lactate, glucose, and PO_2_ levels in the rat hippocampal dentate gyrus during a series of 5 s electrical stimulations (arrows) of the perforant pathway at 2 min rest intervals (reproduced with permission from Hu and Wilson, 1997 copyright, Blackwell, Oxford). The changes in the mean concentration of glucose were always in opposite direction to the changes in mean lactate concentration. The vertical lines were drawn to indicate the simultaneous dip in all three analytes in response to each of the electrical stimulations. For additional details see Hu and Wilson (1997) and Schurr and Gozal (2011) from where the figure and the legend have been reproduced with permission.

Hu and Wilson (1997) published their studies on the coupling of a temporary local energy pool to neuronal activity in the rat brain (**Figure 3**). They were the first to combine the use of three separate sensors (electrodes) with rapid response to measure oxygen, glucose, and lactate. They used them in the dentate gyrus of the rat hippocampus and observed their fluctuation following ten consecutive electrical stimulations of the perforant pathway (a 5 s duration of electric stimulation every 2 min). As has been indicated before (Schurr and Gozal, 2011; see **Figure 3**, which is reproduced here as it was presented upon its first analysis), Hu and Wilson (1997) interpretation of their findings gained both supporters (Pellerin and Magistretti, 2003; Franconi and Merle, 2004; Kasischke et al., 2004; Aubert et al., 2005; Medina and Tabernero, 2005; Serres et al., 2005; Schurr, 2006) and detractors (Dienel and Hertz, 2005; Fillenz, 2005; Korf, 2006). The supporters believed the findings strengthen the proposal that lactate is the monocarboxylate utilized aerobically upon neuronal activation. The detractors disagreed with this conclusion. It should be helpful, for two reasons, to revisit Hu and Wilson (1997) results and reanalyze them beyond the analysis done before (Schurr and Gozal, 2011). First, two decades have passed since the publication of Hu and Wilson (1997) paper, time in which numerous studies added much support to the idea that lactate is a mitochondrial oxidative energy substrate. Second, many other studies on cerebral energy metabolism continue to conclude that neural activity is supported by “aerobic” glycolysis and not by oxidative utilization of glucose, while ignoring the possibility that such activity is supported by oxidative utilization of lactate.

## Revisiting CMR_glucose_ and CMR_O_2__ Following the Paradigm Shift of Glycolysis

Although numerous studies of CBF, CMR_glucose_, and CMR_O_2__ have been published over the years, for the purpose of this review only three studies were selected. The seminal study by Fox et al. (1988), the study by Hyder et al. (1997), the results of which are in disagreement with the former, and the study by Hu and Wilson (1997), the latter mainly because it was the first study to seriously consider the possibility that lactate is a cerebral substrate for oxidative energy metabolism during activation.

**FIGURE 4:**
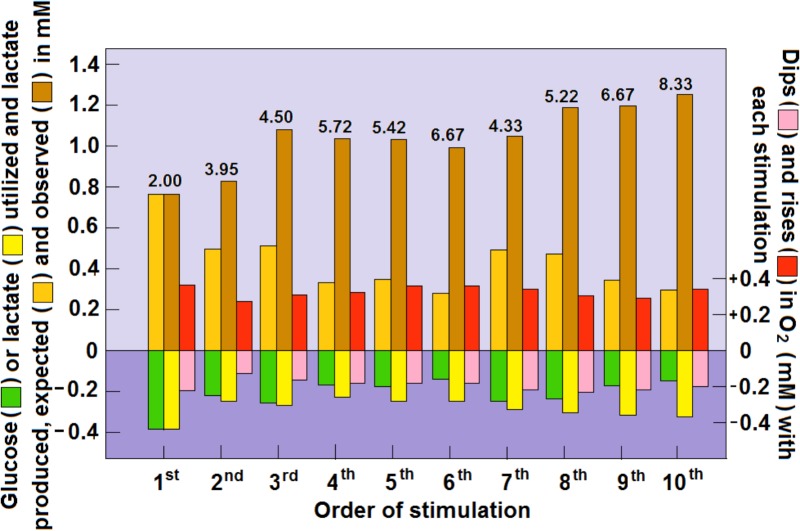
Dynamic relationships of local extracellular glucose, lactate and O_2_ levels in the rat hippocampal dentate gyrus during a series of 5 s electrical stimulations of the perforant pathway at 2 min rest intervals. The concentrations of glucose, lactate and O_2_ were calculated from their dips and rises as measured by Hu and Wilson (1997) using rapid response sensors in their original study. The numerical values above the columns representing the rises in glucose and lactate post-stimulation are the calculated ratios between the two. For additional details see **Figure 3** and Schurr and Gozal (2011).

Our earlier analysis (Schurr and Gozal, 2011) of Hu and Wilson’s findings indicated that upon consecutive stimulation of the rat hippocampal perforant pathway a decrease in glucose consumption was accompanied by an increase in lactate consumption. Moreover, if one is to use the prevailing assumption that glycolytic ATP production is sufficient to fulfill the necessary energy requirements (Fox et al., 1988), in this case those of the stimulated hippocampal dentate gyrus. Apparently, these requirements appeared to diminish with each stimulation or stayed the same at a very low level of 0.8–0.3 mM of ATP production. In contrast, when lactate oxidative consumption is assumed to be the source of the ATP that supports the needs of the stimulated tissue, as lactate consumption increased with each stimulation, so did the ATP production, from 3 mM in response to the first stimulation to almost 11 mM in response to the 10th stimulation. Now, upon further analysis, it is clear that the increased levels of tissue lactate after each stimulation, as measured by Hu and Wilson (1997), could not originate only from glycolytically consumed glucose (**Figure 4**). Clearly, additional lactate had to be recruited from other sources, such as from the surrounding tissue or from glycogen stores (Chambers et al., 2014). As illustrated in **Figure 3**, an increased amount of lactate was consumed during each consecutive stimulation, while the amount of glucose consumed was decreased. Moreover, after each stimulation, except the first one, the amount of accumulated lactate measured was greater than the expected amount that would have resulted from glucose glycolytic consumption, i.e., two moles of lactate per mole of glucose. Following the second stimulation, the tissue ratio of lactate to glucose was 3.95 and following the 10th stimulation this ratio rose to 8.33 (**Figure 4**). Meanwhile, oxygen levels dipped and rose as expected during and after each stimulation, respectively, indicating that the stimulation induced an oxidative consumption of substrate. Initially, glucose and lactate were consumed oxidatively at equal amounts, but from the second stimulation onward more lactate than glucose was consumed. Immediately following each stimulation, a spike in tissue oxygen level was measured, not only assuring that sufficient oxygen is available if needed, but also indicating that the tissue was well oxygenated during the duration of the experiment. It is important to point out that 1 mol of lactate consumes only one half the amount of oxygen (3 mol) for its full oxidation as compared to the amount that 1 mol of glucose consumes (6 mol) for full oxidation. Therefore, if lactate, rather than glucose, is the main energy substrate during neural tissue activation, the expected ratio CMR_O_2__:CMR_lactate_ should not exceed 3:1. Thus, it is reasonable to deduce that under conditions of neural activation, where lactate oxidation is responsible for supplying significant part of the ATP needed to support that activation, the ratio CMR_O_2__:CMR_glucose_ would be significantly lower than 6:1. Consequently, studies where that ratio is calculated to approach 6 (Hyder et al., 1997), the calculators rely on the assumption that aerobic glycolysis produces pyruvate as its end-product, all of which enters the mitochondrial TCA cycle. The opposing conclusions of Fox et al. (1988) to those of Hyder et al. (1997), where the former argue for an almost complete uncoupling between glucose and oxygen consumption of the activated neural tissue, while the latter contend that this coupling is fully maintained, are confusing. Ironically both studies and the interpretation of their outcomes rely on the original, classical paradigm of glycolysis.

While the measurements of glucose and lactate concentrations performed by Hu and Wilson (1997) clearly support the proposed role of lactate as a major oxidative substrate during increased energy needs of activated neural tissue, the relatively small fluctuations in O_2_ levels in response to such activation, measured by polarography, require further consideration. For one, the direct measurement of O_2_ polarographically provides better spatial and temporal resolution and better characterization of the time-course of oxygen responses then BOLD fMRI (Bentley, 2014). The latter method was used by Hyder et al. (1997) and even more cumbersome measurements were employed by Fox et al. (1988), involving the use of [^15^O]H_2_O, [^15^O]O_2_, and [^15^O]CO_2_. Since BOLD fMRI estimates produced a CMR_O_2__:CMR_glucose_ ratio of 6:1, while the ^15^O measurements produced a ratio of 0.4:1, one is left wondering if those measurements and the following calculated values of CMR_O_2__ actually reflect changes in molecular oxygen used during neural activation. Could the direct measurements performed by Hu and Wilson (1997) be somehow reconciled with the indirect ones made by Fox et al. (1988) and Hyder et al. (1997), to provide a more accurate picture of CMR_O_2__, CMR_glucose_, and CMR_lactate_ of activated neural tissue? Where energy (ATP) production of the normal resting brain is concerned, it is widely accepted that over 90% of it originates from glucose oxidation (Siesjo, 1978; Fox et al., 1988). Moreover, since normal glucose concentration in the brain is approximately 2 mM and the normal lactate concentration is about half of that of glucose, it is safe to assume that the normal resting brain is supplied with ample amounts of oxygen to continuously oxidize more than 90% of the brain glucose. In contrast, the glucose supplies to the normal brain are limited (only 40% of normal blood glucose level). The increase in the rate of CBF along with the increase in glucose consumption upon activation (Fox et al., 1988; Ueki et al., 1988; Hyder et al., 1997) should provide all the oxygen necessary to catch up with the increased demand, unlike the limited supplies of glucose. In essence, measurements of oxygen concentrations using low resolution methods are unable to trace local fluctuations accurately if at all, which could explain how Fox et al. (1988) reached the interpretation of their findings. Nevertheless, the conclusion that energy demands of activated neural tissue are fulfilled via glycolytic ATP production is most likely erroneous. In other words, undetectable or slightly detectable dip in tissue oxygen level upon activation is not necessarily an indication that oxygen is not consumed. The higher resolution of oxygen measurement afforded by polarography exemplifies the fact that local oxygen levels dip upon stimulation and overshoot upon its cessation (Hu and Wilson, 1997; **Figures 3**, **4**). Additionally, although local fluctuations in tissue oxygen levels were detected, overall tissue oxygen concentration did not significantly change, perhaps even stayed somewhat above the baseline level. In contrast, significant changes in both glucose and lactate levels were observed (Hu and Wilson, 1997; Schurr and Gozal, 2011; **Figures 3**, **4**). The synchronized fluctuations in both lactate and oxygen clearly indicate that lactate is being oxidized upon tissue stimulation. Interestingly, during the 20 min period that began after the 10th stimulation, both oxygen and glucose tissue levels appeared to increase above the baseline level, as the high lactate levels gradually declined (Hu and Wilson, 1997; **Figure 3**). If any, these shifts indicate that during recovery post-stimulation lactate becomes the preferred oxidative energy substrate, sparing glucose. The preference for lactate over glucose, especially when the former is abundantly available is understandable, since lactate oxidative utilization, contrary to glucose, does not require any investment of ATP prior to its utilization by mitochondria. Disappointedly, almost two decades after the publication of their study (Hyder et al., 1997) these investigators, with other collaborators, continue to promote the concept that aerobic glycolysis is sufficient, where human brain gray matter is concerned, to supply most or all the necessary ATP for the activated neural tissue (Hyder et al., 2016).

## Conclusion

Whenever a scientific paradigm shift occurs it brings about the reconsideration of hypotheses and concepts that have been formulated according to the foundation on which the older concept was built. Our understanding of CMR_O_2__ and CMR_glucose_ of the resting and activated neural tissue, and the choice of the best methods to measure these rates and interpret the results have always relied on two basic postulates: (1) Cerebral energy metabolism requires the breakdown of glucose via glycolysis and the utilization of its end-product, pyruvate, by the mitochondrial TCA cycle and the electron transport chain with oxygen as its final receptor; (2) activation of cerebral tissue is supported by an increase in ATP production and thus an increase in glucose and oxygen consumption. Two seminal papers, published almost simultaneously (Fox et al., 1988; Schurr et al., 1988), have compelled scientists in the field to reconsider these two basic postulates. The former has confounded many with its conclusion that the energy needs of activated neural tissue are minimal and are answered by glycolysis alone (glucose → lactate + 2ATP); the latter also bewildered many, as it demonstrated the ability of neural tissue to function and be activated when lactate was the sole oxidative energy substrate (glucose → lactate; lactate + O_2_ + mitochondria → pyruvate → TCA cycle → CO_2_ + H_2_O + 17ATP). The idea of lactate as a suitable oxidative energy substrate has gained much support over the past three decades. Glycolysis as a sole supplier of the energy needs of the activated neural tissue is an idea that still divides the scientific community. By accepting the new paradigm of glycolysis and apply it in the interpretation of the results of the studies by Fox et al. (1988), Hu and Wilson (1997), Hyder et al. (1997, 2016) and many others, one can visualize a scenario where lactate plays a major role in providing the necessary energy for the activated neural tissue. Taking into consideration the scientific data and the line of reasoning discussed in this review, a strong argument can be made against the idea that the energy needs of activated neural tissue can be solely provided by the glycolytic pathway or via glucose oxidative metabolism alone. Therefore, it is strongly suggested that future studies of activated cerebral metabolic rates include along with the measurements of CMR_O_2__ and CMR_glucose_ the measurement of CMR_lactate_.

## Author Contributions

The author confirms being the sole contributor of this work and approved it for publication.

## Conflict of Interest Statement

The author declares that the research was conducted in the absence of any commercial or financial relationships that could be construed as a potential conflict of interest.
